# Effectiveness, Esthetics, and Success Rate of Dental Implants in Bone-Grafted Regions of Cleft Lip and Palate Patients: A Systematic Review and Meta-Analysis

**DOI:** 10.7759/cureus.49101

**Published:** 2023-11-20

**Authors:** Ankita Pathak, Mithilesh M Dhamande, Seema Sathe, Smruti Gujjelwar

**Affiliations:** 1 Prosthodontics and Crown & Bridge, Sharad Pawar Dental College and Hospital, Datta Meghe Institute of Higher Education and Research, Wardha, IND

**Keywords:** success rate, bone grafting, dental implants, cleft lip, cleft palate

## Abstract

Congenital clefts impair function and appearance, thus impacting a patient's social and mental health. A multidisciplinary team that can offer comprehensive treatment from infancy through maturity and beyond can successfully address these abnormalities. Dental rehabilitation is very important for these patients; these abnormalities should be identified and must be treated accordingly. Hence it is of utmost importance to know the success rate and changes in quality of life from patient satisfaction in order to plan future treatment goals when coming across such cases. The aim of this article is to determine success rate, esthetics, and patient satisfaction in implant-based oral rehabilitation in bone-grafted regions of cleft patients. Registration was carried out in PROSPERO (International Prospective Register of Systematic Review) with registration number CRD42022329861 on May 7, 2022. Medical Subject Headings (MeSH) terms such as cleft lip, cleft palate, survival rate, and dental implants were used to handpick articles via an electronic database. Preferred Reporting Items for Systematic Reviews and Meta-Analyses (PRISMA) guidelines were followed to compile all the data gathered from an electronic database. According to the collected data, 93.5% of the survival rate with dental implants was noted irrespective of the type of bone grafts used. Also, improvement in quality of life was achieved in these patients. Effectiveness, esthetics, and success rate are very well achievable with implants in cleft patients. Although the aesthetics are not equivalent to that of dental implants in normal patients, patient satisfaction is still satisfactory in patients with dental implants in bone-grafted regions of cleft lip and palate.

## Introduction and background

Cleft lip and palate (CLP) are genetically predisposed developmental defects. These anomalies were discovered through inherited genomic mapping. Orthodontists play a wide range of roles, starting with infant orthopedic nasoalveolar molding and continuing through adolescence. Orthodontic space closure with concurrent aesthetic restorative contouring is the preferred therapy [1]. Orthodontic therapy in cleft individuals achieves only 50% to 75% closure of the residual gap, necessitating dental prostheses to close the remaining space [2-3]. A cleft is often surgically treated with bone grafting, which supports the eruption of canines, allows for orthodontic tooth movement, and provides an opportunity for planning and placing implants. In particular, the iliac bone is regarded as the gold standard for this reconstruction as it provides strong mechanical strength for fixation stabilization and a higher potential for osteogenesis [4].

Reconstruction is often performed during the mixed dentition stage when 2/3 of the root of the canine is developed. Since individuals continue to develop until early adulthood, surgical treatment with implants is not the ultimate viable choice for repairing midline diastema in previous cleft cases [5]. Fixed partial dentures and removable partial dentures (FPDs and RPDs) should be considered where implant placement is not possible. However, each of them has drawbacks, especially concerning tooth wear and cosmetic flaws [6]. The absence of a papilla and scarring of the soft tissue can cause esthetic (black triangle) and phonetic (air leakage) issues. In reaching a satisfactory esthetic result, optimal three-dimensional implant positioning is crucial, but it is also known that a good esthetic outcome is the result of the combination of harmonious teeth, gingival appearance, and lip shape.

The gingival tissue is of fundamental importance because the quantity and quality of the keratinized gingiva around the prosthetic abutments and implants creates a barrier against inflammation and facilitates oral hygiene [7]. Optimum three-dimensional implant location is crucial in achieving a desirable esthetic result. Dental implant-based rehabilitation provides a suitable solution; however, its success depends on the quality and quantity of the residual bone. Hence, it is important to know what will be the success rate and esthetics of dental implants in bone-grafted regions of cleft lip and cleft palate [8]. The goal of this systematic review was to determine the esthetics, efficacy, and success rate of dental implants in syndromic CLP patients. It also aimed to list various grafting sources and the likely outcomes associated with other criteria indirectly contributing to assessing esthetics, success rate, and patient satisfaction.

## Review

First, the 27-item Preferred Reporting Items for Systematic Reviews and Meta-Analyses (PRISMA) checklist was used to conduct the current systematic review [9]. The current review was conducted by following the patient population, intervention, comparison, and outcome (PICO) standards [10]; where P: unilateral and bilateral CLP patients with missing permanent teeth in the cleft region; I: dental implant-based rehabilitation in bone-grafted regions of cleft lip and cleft palate; C: no comparison groups; O: success rate, esthetics, and patient satisfaction of dental implants in bone grafted regions of CLP patients. Final research question: what are success rate, esthetics, and patient satisfaction in patients rehabilitated with dental implants in bone-grafted regions of cleft lip and cleft palate patients? The systematic review was carried out by predefined analytic, exclusion, and inclusion criteria and was filed with the International Prospective Register of Systematic Review (PROSPERO) under the registration number CRD42022329861 (Record ID: 329861).

**Figure 1:**
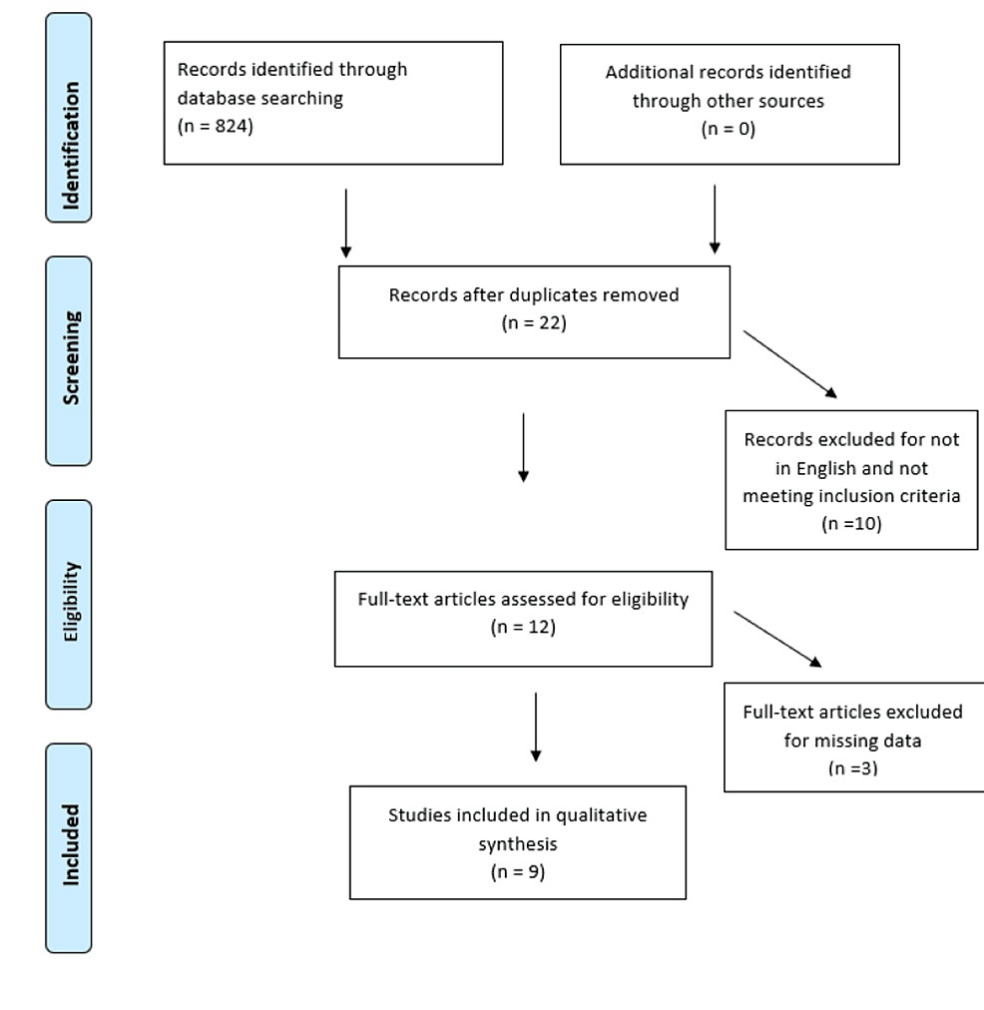
PRISMA Flow chart showing the selection of studies.

The data extraction and search strategy were carried out through electronic databases such as PubMed, Google Scholar, Cochrane Library, Latin American and Caribbean Health Sciences Literature (LILACS), Web of Science, etc. MeSH terms dental implants, cleft lip, and cleft palate were used in an advanced search. Filters were applied for English language and human studies. Inclusion criteria included the average age of patients 21 years old, articles published in the English language, prospective and retrospective studies, randomized control trials, and the utility of implants for dental rehabilitation in cleft patients. Exclusion criteria were incomplete studies, incomplete and unpublished randomized control trials, letters, editorials, abstracts only, animal studies and in-vitro studies, cleft associated with syndromes, and papers or studies in which clinical parameters were not discussed. Figure 1 shows the PRISMA flow chart showing the selection of studies for the review and meta-analysis. Table 1 shows a summary of the inclusion and exclusion criteria. The Joanna Briggs Institute (JBI) critical appraisal tool was used to reduce the risk of bias. Two reviewers independently examined the aforementioned available literature, and selection criteria & exclusion criteria were documented using the JBI tool [11]. In the event of a dispute, a third party requested an independent assessment article in question, and the item was included or omitted based on a majority judgment.

**Table 1:** Inclusion and exclusion criteria summarised as above mentioned in the table.

Criteria	Articles
Inclusion criteria	Average age of patients 21 years old. Articles published in the English language. Prospective and retrospective studies, randomized control trials. Utility of implant for dental rehabilitation in cleft patients.
Exclusion criteria	Incomplete studies, incomplete and unpublished randomized control trials. Letters, editorials, and abstracts only. Animal studies and in-vitro studies. Cleft associated with syndromes. Papers or studies in which clinical parameters were not discussed.

Results

A total of nine studies were included for qualitative synthesis of data and two studies for meta-analysis of systematic review. A total of 308 implants were placed; implants were placed on a total of 227 cleft patients (an average of 1.31 implants per patient) in the included studies. The mean age of the patients was 21 years. An average survival rate of 93.5% was extracted from the studies. According to the included studies, the follow-up period is six months to five years. As per the reported literature, the anterior iliac crest is the gold standard site for bone grafts in cleft patients. In the reported literature by Leven et al., in cases such as atrophic maxilla, where dental implants are not the ultimate treatment choice in these patients, zygomatic implants are considered. He concluded in his study that a high success rate can be achieved when dental implants are replaced by zygomatic implants [12]. Table 2 shows included studies in the present systematic review​​​.

**Table 2:** Included studies in the present systematic review IPS: patient-specific implant; IABH: interdental alveolar bone height

Reference	Year of publication	Methods	Participants	Interventions	Bone grafting	Control	Outcomes	Survival rate
Leven et al. [12]	2022	Prospective study	Alveolar cleft patients, n=7	Zygomatic implants, n=17, dental implants, n=8	Autogenous bone scrapings, xenograft	Nil	Success rate	100%
Landes et al. [13]	2012	Retrospective study alveolar	Alveolar cleft patients, n=17	Dental implants, n=24	iliac crest bone	Nil	Mobility, probing depth, plaque–index and peri-implant bleeding index	95.8%
Takahashi et al.[14]	2008	Prospective case series alveolar	Alveolar cleft patients, n=16	Dental implants, n=23	iliac crest	Nil	IABH	100%
João Luiz et al.[15]	2018	Retrospective case series alveolar	Alveolar cleft patients, n=93	Dental implants, n=120	iliac crest, mentum	Nil	Functional effectiveness	94.2%
Van Nhan et al. [16]	2018	Prospective clinical trials	Alveolar cleft patients, n=32	Dental implants, n=32	Iliac bone	Nil	Bone formation using Enemark scale, implant health using Misch criteria	100%
Surin et al. [17]	2020	Retrospective case series alveolar	Alveolar cleft patients, n=8	Dental implants, n=12	autogenic in three patients and alloplastic in five patients	Nil	Periodontal clinical and radiographic examination	91.7%
Alberga et al. [18]	2020	Case-control study	Alveolar cleft patients, n= 17	Dental implants, n=24	Anterior iliac crest bone	17 matched control group	Marginal bone loss, esthetics , patient satisfaction	95%
Savoldelli et al. [19]	2022	Retrospective study	Alveolar cleft patients, n=40, 26 patients treated with dental implants	lmplants, n=40	Anterior iliac crest	14 patients closure or no rehabilitation	Assesment of marginal bone loss with novel method	90%
Ralf et al. [20]	2022	Retrospective study	Alveolar cleft patients n=6	IPS preprosthetic implants, n= 11	Nil	Nil	Success rate	100%

Result of meta-analysis

**Figure 2:**
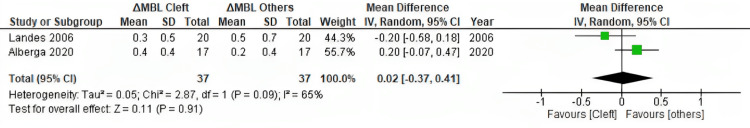
Forrest plot comparing mean MBL in cleft patients. SD: standard deviation; MBL: marginal bone loss Landes 2006: Landes et al., 2006 [13]; Alberga 2020: Alberga et al., 2020 [18]

**Figure 3:**
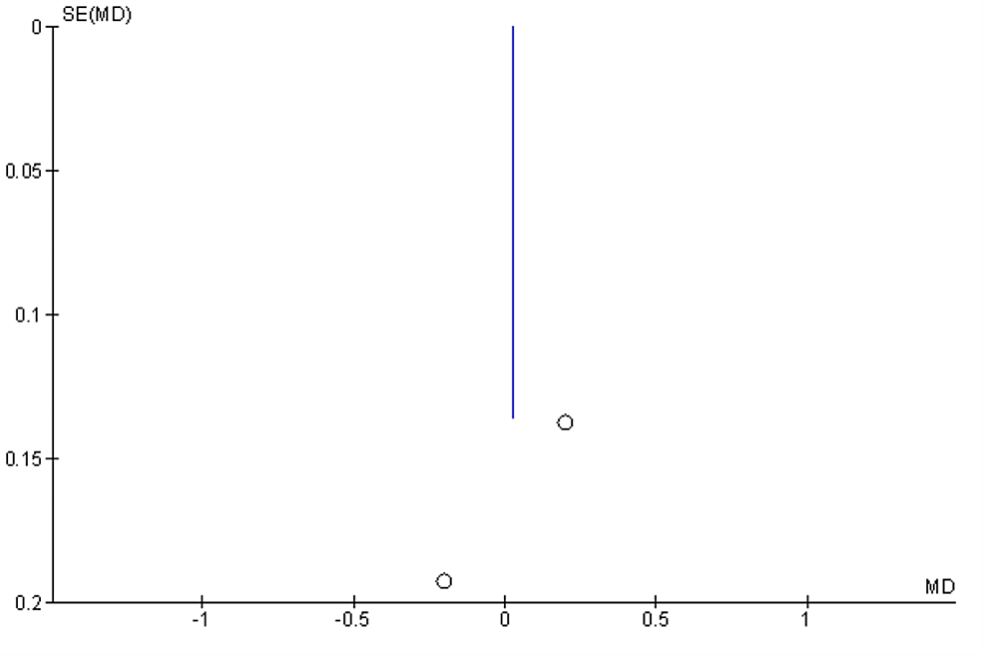
Funnel plot showing MBL in cleft patients and other patients in follow-up after implant SE: standard error; MD: mean difference; MBL: marginal bone loss

The result of the Meta-analysis of the two studies suggests no significant difference in the change of marginal bone level in the implant area in both the cases and the control, suggesting equal effectiveness of the implant in both cleft and non-cleft groups. (Random-effects: difference in means = 2.87, 95% CI= -0.37 to 0.041; p = 0.09). The result of the individual study suggests no significant difference in the marginal bone level -0.04±0.04 in the cleft group and -0.02±0.04 in the control group. Similarly in the other study, 0.03±0.05 and 0.5±0.07 were in the cleft group and the control group, respectively. Bone loss is also seen in other studies, but it didn’t affect the integrity of the implants. Study confirms that the success rate of the implants is achieved when bone loss is less than 1.5 mm and 1.9mm [21-23]. Figure 2 shows the results of the meta-analysis, viz., a forest plot comparing mean marginal bone loss (MBL) in cleft patients. Also, a funnel plot (Figure 3) shows MBL in cleft patients and other patients in follow-up after implant. 

Most of the studies included in the systematic review performed bone grafting using either autogenous iliac crest (AUIC) or allergenic bone grafts, which have shown excellent outcomes in managing cleft patients [12-20].

Discussion 

The existing database emphasizes the multiple benefits of dental implants in CLP patients [12-20]. Other parameters assessed by the authors included in the review are summarised in Table 3.

**Table 3:** Other criteria

Author	Landes et al. [13] 2006	Takahashi et al. [14] 2008	João Luiz et al. [15] 2018	Van Nhan et al. [16] 2018	Surin et al. [17] 2020	Alberga et al. [18] 2020	Savoldelli et al. [19] 2022	Leven et al. [12] 2022	Ralf et al. [20] 2022
Clinical/periodontal criteria									
Bleeding index	_					+			
Gingival index	+								
Plaque index	+				+	+			
Probing depth	+					+			
Gingival/mucosal recession	+								
Width of keratinized gingiva	+				+				
Insertion torque									
Implant mobility	+			+	+				
Suppuration at the implant site				+	+				
Pain				+					
Pink and white esthetic scale									
	+					+			
Radiographic criteria									
Cone beam CT (CBCT)			+		+		+	+	+
Enmark Scale				+					
Radiolucency at implant site periapically					+				
Marginal bone loss (MBL)		+	+	+	+	+	+		
Interdental alveolar bone height (IABH)		+					+		
Patient satisfaction									
Self-administered questionnaire	+					+			
Implant crown esthetic index							+		
Other parameters									
				Assessment of fistula formation			Novel method to assess marginal bone levels.	Palatal fistula present in six patients	Oronasal fistula

As given in the literature by Takahashi et al., 2008, the evaluation of dental implants is done by the assessment of alveolar bone height [14]. Their study, as well as the study done by Savoldelli et al., 2022, used the interdental alveolar bone height (IABH) index for the assessment of bone height, as shown in Table 4. In the two studies, the success rate of implants was 90% and 100%, respectively [14,19].

**Table 4:** IABH score IABH: inter alveolar bone loss

Score	Bone loss
4	0%-25%
3	25%-50%
2	50%-75%
1	75%-100%

Achievement of Considerable Esthetics After Rehabilitation With Implants

In terms of esthetics, it’s well understood that soft tissue scars and an absence of interdental papilla can lead to less-than-desirable esthetic results [24-28]. Although the optimal 3D placement of the implant is important for attaining a desirable cosmetic result, it’s understood that a good esthetic outcome is the result of a combination of harmonious teeth, the ultimate zenith of gingiva and healthy gingiva, and perfect lip line and contour [7]. Enhancements in esthetics boost the patient’s self-perception and improve quality of life [28-30]. These lower scores are most likely the outcome of a less favorable preoperative condition, including the formation of scar tissue. In patients with an alveolar cleft, the implant placement is frequently accentuated, putting them at risk of less desired cosmetic consequences.

Patient Satisfaction 

To determine patient satisfaction, a self-administered questionnaire and an implant esthetic crown index were used. The implant crown esthetic index (range 0-5) was used to assess patient satisfaction. Compared to the pink aesthetic score, deemed overly strict for CLP patients [31], the implant crown aesthetic index was well-matched for patients with CLP.

Factors Considered in Determining Therapeutic Success of Implants

The notion of implant success has evolved throughout the field of implantology. Unlike past philosophies that emphasized a single component as the driving factor for success, the present philosophy regards the implant-prosthetic complex as a single entity, valuing clinical and radiological criteria, prosthesis, esthetics, and function equally. In the current review research, there was a lack of mutual agreement on documented characteristics to determine implant success. Although disputed, the breadth of connected gingiva is frequently seen as a crucial determinant of implant success. Although the value of connected gingiva does not determine a patient's capacity to maintain hygiene, lower values have been demonstrated to cause higher plaque formation, irritation, bleeding of the gingiva, and periodontal problems, all detrimental to implant health [31,32]. Upto 2 mm or larger of gingiva is assumed adequate for peri-implant health. After analysis, every author agreed that there was an improvement in aesthetics and soft tissue profile [33].

Analysis of the Success Rate of Placed Implants

A success rate of 95% to 100% is achievable as per the available literature, except for case studies for assessing implant success. Therefore, a high success rate can be observed along with bone grafting in the cleft regions, showing that it is frequently linked with a significant risk of complications [34]. According to the study, implants in the anterior region of the maxilla showed a 2.1% - 6.2% failure rate [35,36]. Implants in the maxillary region failed at a much greater incidence than those in the mandible when subjected to rapid stress. In type III bone, a higher failure rate was observed, i.e., 3% in the anterior region of the maxilla, which may explain the relatively high failure rate in the anterior region of the maxilla. Follow-up periods vary from six months to 40 months [37].

Limitation 

There are very less studies including intervention as well as control groups. Due to this, less literature is available for meta-analysis. A low level of evidence is available to generate a meta-analysis. Therefore, more randomized control trials should be conducted in the future.

Future direction 

Retrospective and prospective studies are included in the present systematic review. The majority of studies contain only intervention groups only. However, more randomized control trials should be performed to highlight the effectiveness and survival rate of implants in cleft patients.

## Conclusions

Patient satisfaction is comfort to the patient, and comfort to the patient is a token of appreciation to the clinician. The longevity of implants in cleft patients has reported a high survival rate in the literature database. The negligible difference in bone loss compared to other cases shows great success. In a correct graft, the optimum gap between the implant placement and selection of implant provides a high success and survival rate. This systematic review intended to understand and explore the survival rate of implants in cleft regions. A major finding from the included literature highlights no significant change in MBL in cleft and non-cleft patients. This data strongly suggests equal effectiveness of implants in both groups. However, more clinical trials are required in this field to buttress the evidence.
